# Reinforcing the foundations of ornithological nomenclature: Filling the gaps in Sherborn’s and Richmond’s historical legacy of bibliographic exploration

**DOI:** 10.3897/zookeys.550.10170

**Published:** 2016-01-07

**Authors:** Edward C. Dickinson

**Affiliations:** 1 Flat 3, 19 Bolsover Road, Eastbourne, East Sussex, BN20 7JG, U.K.

**Keywords:** Ornithology, stability, priority, ICZN Code, dates of publication, ZooBank, verification, LANs, family-group names, genus-group names, species-group names, taxon-sampling, synonymy, objective synonyms, subjective synonyms

## Abstract

Due to its public popularity, ornithology has a huge corpus of scientific publication for a relatively small number of species. Although there are global checklists of currently recognised taxa, there has been only limited, mainly individual, effort to build a nomenclatural database that the science of ornithology deserves. This is especially true in relation to concise synonymies. With the arrival of *ZooBank* and the *Biodiversity Heritage Library*, the time has come to develop synonymies and to add fuller bibliographic detail to databases. The preparation for both began at the start of the 20^th^ century with extensive work by Sherborn and Richmond. I discuss their legacy, offer notes on significant work since then, and provide suggestions for what remains to be done. To make solid the foundations for ornithological nomenclature and taxonomy, especially for synonymies, ornithologists will need to collaborate much more and contribute to the digital infrastructure.

## Introduction

As an old and popular science, ornithology has a very substantial literary foundation. Linnaeus (1758, 1766) provided the starting point, and the elaborated rules for scientific nomenclature meant that the content of the ornithological literature can be organised and is capable of providing detailed histories of our understanding of each avian taxon. Such histories are easiest to compile if names have not changed. However names do change, especially when a species is re-interpreted to belong to a different genus with the addition of new data or changing taxonomic perspectives. The Linnaean binomial (binominal) system provides for such changes in our understanding of relationships first, by allowing for new genus-group names to be introduced and second, in the context of the required combination of two names – the genus-group name and the species-group name – by maintaining the species-group name when transferred, subject only to gender agreement. Linnaean nomenclature is rooted in Latin and Greek, although it has been enriched by the acceptance of names from other sources. Because of these classical roots and the worldwide convention of acceptance of these rules, scientific names form the *lingua franca* of the world’s zoologists. Ornithologists in Morocco, Japan or the United States of America understand the name *Eremophila alpestris* to refer a species of bird that is consistent across their language and geographic differences.

To be nomenclaturally ‘available’ in the technical sense, (i.e., validly published according to accepted nomenclatural rules) the name when first used must have been the first applied to the taxon after 1757 in binominal format (i.e. in a combination of genus-group name and species-group name). Thus, first use implies that the date it is introduced determines whether it gains priority and can be used. Other names may be available but date precedence will normally dictate which name should be chosen – the oldest name should be used according to the Principle of Priority in the *International Code of Zoological Nomenclature* (current edition, ICZN 1999; hereafter ‘the Code’). This approach, set out as a declared Principle (or foundational rule) is thought to have been taken from early doctrine in patent law. Thus, the determinant evidence for each contending name includes the publication date along with the name of the author. The decision on priority may potentially rest on the very day of publication.

However, if a name is later used in a different combination due to assignment to a different genus, homonyms (identical names, in this case at the species-group level) must be resolved. Any other taxon found within the newly relevant genus that already bears the same specific or subspecific name can prevent the retention of the original species-group name. This can happen surprisingly often, as species-group names may refer to relatively common characteristics (e.g., *alba* for white, *atlantica* for distribution, etc.). To be retained, the transferred name must be older than any contending name. In any such case one of the two homonyms must be discarded and a replacement name found from the list of available synonyms if possible, or be freshly established if necessary. Making this evidence available is vital for understanding the logic behind historical name changes.

Sherborn (1861–1942) recognised the importance of this evidence for maintaining sense in the shifting meanings of the world’s diversity. His creation of a card index, which, when sorted, became the *Index Animalium*, is remarkable both for the size of the task he set himself and for his years of application to that task for minimal reward (Evenhuis 2016, Shindler 2016, Taylor 2016, Welter-Schultes et al. 2016). The *Index Animalium*, which ran to almost 9600 pages, has been scanned and made available through the 'Smithsonian Libraries' website (http://www.sil.si.edu/digitalcollections/indexanimalium/ see Pilsk et al. 2016). In so far as ornithology is concerned this work provided a near-complete, and very largely accurate, dataset of bird names from 1758 to 1850. It should be emphasised that this index of old names provides the foundation, and thus is the most critical basis, for a stable modern nomenclature and links to past published scientific information. In compiling *Index Animalium* Sherborn worked first on the period 1758 to 1800 (Sherborn 1902) with the library resources of the Natural History Museum in London (NHM-London) at his disposal, doubtless working all the way through each volume in turn. He then tackled 1801 to 1850 in a 33-part work appearing from 1922 to 1935. His card index is held in the Rare Books Room at the NHM-London and remains of value as some cards reveal more than Sherborn included in his one-line entries in the *Index*.

Sherborn’s work came to the attention of Charles Wallace Richmond (1868-1932) at the Division of Birds at the National Museum of Natural History (NMNH, a part of the Smithsonian Institution Washington, D.C.) who had begun a card catalogue of bird names by 1896, while working closely with his mentor, Robert Ridgway, who was writing the *Birds of North and Middle America*. By then Richmond had been collecting such names since around 1889 (Stone 1933).

**Figure 1.**
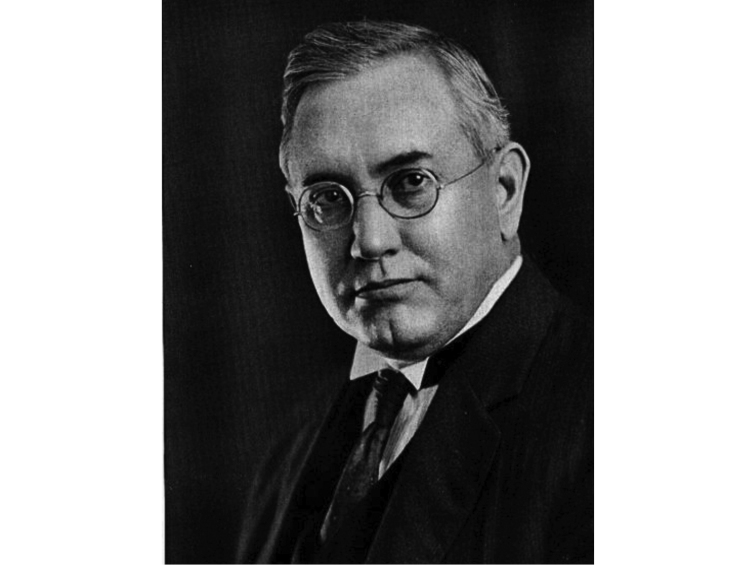
Charles Wallace Richmond as depicted in his obituary in *The Auk* by Stone (1933).

Richmond, whose card index related solely to birds, made it his business to build his content well beyond the 1850 date reached by Sherborn, and this task was taken up by those who followed him in the NMNH. He was, or became, just as interested in the importance of dates of publication as Sherborn and the two corresponded on this topic. Richmond was very determined in his search for avian genus-group names and his card index of these became the basis for four supplements to the *Index Generum Avium* of Waterhouse (1889) (Richmond 1902, 1908, 1917, 1927) – and these often included names that had escaped both Waterhouse and Sherborn.

The Richmond Index, published in microfiche form in 1992, sixty years after Richmond’s death, was a relatively comprehensive reference system when Richmond last worked upon it. Just how comprehensive is unclear as Olson and Browning (1992) offered the caveat that “Richmond died in 1932 but his contributions to the Index had probably diminished well prior to that. Stone (1933) relates that Richmond’s health began to fail about the onset of the First World War, after which his work on the Index was only desultory”. Thus there may be significant gaps during a period from about 1914 to the 1930s. Over the sixty years between Stone’s death and publication, there was a clear recognition of its utility by the Division of Birds, NMNH, which held the resource in its library. Smithsonian staff did much to add to it. However, again a caveat is in order from Olson and Browning (1992), who wrote “this was attended to with varying degrees of competence and dedication. More diligence has been applied in recent years, but there remains a period for which the *Index* is certain to be recognizably incomplete”. Nonetheless, the list can reasonably claim to be more complete than any other for birds available today.

The Richmond Index includes both genus-group name cards and species-group name cards. These were microfilmed and appeared in microfiche form (Richmond 1992). More recently these cards have been scanned by Alan Peterson and made available on www.zoonomen.net. Richmond also used index cards to record his research into the dates of publications, both part-works and journals and to keep notes about authors. These were very much works-in-progress, and have not been published; they are housed in the library of the Division of Birds, NMNH.

Sadly Stone did not tell us precisely when Richmond began to correspond with Sherborn saying “through all these years and up to the time of his death Richmond maintained a correspondence ...” not only with Sherborn, but also “with Gregory Mathews and others interested in bibliographic research”. But the start of their sharing of their findings surely cannot have been later than 1902 when the first part of the *Index Animalium* appeared. Letters kept by Sherborn are archived in the Palaeontology Library NHM-London, but these still need to be explored in depth.

Thus, when Sherborn died in 1942 the primary sources for avian names were the *Index Animalium* up to 1850 and, up to and beyond that date, the Richmond card index although it was not put in wider circulation for another 50 years. My aim with this chapter is to define the gap between what they left us and what we ought now to have, and to discuss the extent to which that gap has now been closed. To do this I need first to suggest what I believe we should have available to us. Then, I will record some of the major works in ornithology which have filled large parts of that gap. And, finally, I will offer a summary of what remains to be done.

## The organised resources that the ornithological community needs:

A comprehensive set of all validly published avian scientific names complete with their authors, dates and citations. This dataset should consist of original combinations with original spellings, right or wrong. Where introduced with dual or multiple spellings, each such spelling should be in the dataset and information on the subsequent selection of a spelling as correct by a First Reviser should be located and a citation to that added [Art. 32.2.1, ICZN Code]. First Revisers have a special definition and value in nomenclature [defined in the ICZN glossary as “The first author to cite names (including different original spellings of the same name) or nomenclatural acts published on the same date and to select one of them to have precedence over the other(s).” and supported in Art. 24].

The structure of that dataset should link every name that first appeared on the same date in the same work thus allowing any date change to cause change in each linked record. Also relevant will be any published First Reviser action in which precedence of one work rather than another has been asserted because this affects the dates of publication of record for both publications and may affect more than just the specific case dealt with by the First Reviser (so the record for each such work should hold a notation of this kind).

Fully functional nomenclatural synonymies need to be organised. Not synonymies of the kind found in 19^th^ century works like the *Catalogue of the birds in the British Museum* where the objective was to list every use of a name listing each of its combinations and spellings – although such synonymies have value in a different context. Instead, a nomenclatural synonymy needs to show the relationships between senior and junior names for the same taxon. In the context of genera any names of subgenera must be included as related subordinate names and in the case of species the subspecies and their synonyms must be included. At genus-group level, where phylogeneticists could immediately benefit, the broad genus would have in its synonymy all the subgenera and the synonyms that relate to the broad genus name. These should be qualified according to whether they may be objective synonyms – based on the same type species – or subjective synonyms which, in the right circumstances, taxonomists might choose to bring into use. At the species group level where, in principle, each name is based on a type specimen there are again objective synonyms (based on the same type specimen) but more often synonyms at this level are subjective. Due to taxonomic change these synonymies would need periodic review because subjective synonyms might well come into use. There are tools available today to help maintain such lists and some branches of zoology, such as icthyology and several sections of entomology, have established networks of scientists who have committed to help with such tasks. Ornithology, having been judged to be “now well-known” for over 50 years, is finding that it needs to catch up!

The *Code* requires that account be taken of other related issues. Thus the main dataset should include, or be linked to, full information on the approved changes to original spellings. This, with the possible exception of changes due to gender agreement (Art. 32.3) as they can be dynamic, implies: (i) corrected spellings as governed by Art. 32.5 (with retention and signalling of an incorrect original spelling because that is what a researcher may find when checking the original), (ii) justified emendations as governed by Art. 33, or mandatory changes as defined in Art. 33 and (iii) the need for a notation as to the chosen original spelling selected by a First Reviser (Art. 32.2.1) from two or more original spellings. Finally, notes must be added regarding any decisions made by the Commission that fix a spelling (see ICZN 1987, 2001).

*ZooBank* (http://zoobank.org), the registration platform of the International Commission on Zoological Nomenclature, was conceived not just to hold names but also nomenclatural acts – although progress towards accommodating such acts has so far been limited. The Code places central importance on such acts and implicitly requires that a collection be made of all such acts thus the establishment of *ZooBank* is the logical outcome of the Code and the shift to digital taxonomic tools. The extent to which any zoological discipline has compiled such lists is unclear but it is not easy to find any such lists made for ornithological cases. Centralisation makes sense, so *ZooBank* is the logical place for confirmation that Code-based requirements have been met and for showing the effects on the original name. The stability of spellings is partly dependent on access to this information, but so too is the avoidance of contradiction of any First Reviser action relating to a spelling choice. Special attention will be needed for recording First Reviser actions that give precedence to one work over another. These actions need association with the first works involved and the discovery of what other names, perhaps in a different discipline, may be affected. Anyone accrediting any zoological or botanical name to that work will need to do the underlying bibliographic research to do the job properly.

Even if the basic information is only partially complete, deposition in *ZooBank* makes any set of names accessible, i.e., immediately retrievable, for a global community of users. Thus importing the names from Sherborn’s *Index Animalium* makes sense although there would then remain the challenges of completing the dataset, verifying the names, and establishing which names are ‘available names’ in the Code-specified technical meaning. *ZooBank* will need to signal for each registered name whether it has been verified, and, where appropriate, to signal which published names have been found to not be ‘available’ in the sense of the Code.

The development of Lists of Available Names (LANs; see Art. 79 of the Code) would be assisted by such information access, which seems to be a necessary preliminary step. In this context see Alonso-Zarazaga et al. (2016).

Some zoological groups, whether taxon-rich or not, lack the mass of serious publication per taxon that is found in ornithology. No current ICZN Commissioner works extensively on ornithological taxonomy and it may be that the value of such a mass of literature is under-appreciated by commissioners not working in fields that are similarly rich in bibliographic history. A thorough understanding of the literature as it relates to any given taxonomic group gives a much stronger qualification for decisions on name availability for that group.

Proposed LANs need to provide an appendix of unavailable names, giving the reason each was decided to be unavailable (Alonso-Zarazaga et al. 2016). There are problems with the suppression of names, not only because they may confound past decisions on homonymy, but also because lack of use on its own is an insufficient reason for suppression – especially at a time when molecular studies are revealing sibling taxa to which some of our ‘unused’ names are being found to apply. Although a reservoir of synonyms should be valued and not considered a problem, it would be reasonable to suppress old names that have not been retained in synonymies. In particular it would be a mistake to suppress genus-group names that may well be available and valid for unrecognised subgenera. By contrast, issues of homonymy aside, names which are objective synonyms may merit suppression although, in this information age, it is hard to see any gain coming from full suppression as opposed to listing as unavailable with potential approval for restoration.

## What help, since the work of Sherborn and Ridgway, has been provided for the building of synonymies?

Neither of these compilers left us fully comprehensive lists of avian names or sought to arrange synonymies at any rank. However, since then the work of Bock (1994) covering family-group names is available. Bock (1994: 13) acknowledged the help available to him from a card index of family-group names which Ernst Mayr had prepared about 1960. Bock’s work, proposed during discussion of the 4^th^ edition of the Code, was intended to pave the way for a List of Available Names for ornithology although unfortunately Bock’s expectations of the Code were not all met in the final drafts of the relevant articles. Indeed until recently (Alonso-Zarazaga et al. 2016) the ICZN had not provided clear guidelines on format or procedure. Some well-considered suggestions were made after Bock’s work appeared and all these elements will need to be taken into account when a submission is prepared for a LAN for avian family-group names, for which Bock’s work provides a very helpful foundation.

At the level of genus-group names help is at hand from general zoological nomenclators. The well-known *Nomenclator Zoologicus* by Sheffield Airey Neave (1879–1961) was published in four volumes (1939–40), and has been complemented by supplements that have continued into the present century and is accessible online (http://uio.mbl.edu/NomenclatorZoologicus/). The less well known, but useful, *Nomenclator Animalium generum et subgenerum* by Franz Eilard Schulze (1840–1921) was issued in parts from 1926 to 1954. One or two ornithologists with a particular personal interest in genus-group names will facilitate the timely preparation of a synonymy of genus-group names if they can be recruited.

As for where names of “missing” new species and subspecies of birds may be found, we have a huge corpus of ornithological literature, and synonymies can be found in many of the more scientific works.

At the global level, for anyone seeking to develop a synonymy of avian names, two works provide the backbone. The first was the *Catalogue of the Birds in the British Museum* (1874 to 1898), totalling 27 volumes, compiled mostly by Richard Bowdler Sharpe (1847–1909). This took the starting point of binomial nomenclature as Linnaeus (1766 – the 12^th^ edition of the *Systema Naturae*) and thus, unfortunately, excluded all earlier references. Acceptance of the 1758 10^th^ edition of the *Systema Naturae* (Linnaeus, 1758) as the start point for zoological nomenclature dates from 1886 in America (Banks 2004), and in Europe apparently only from the *Règles* (ICZN 1905), but the *Catalogue* had been begun with the 12^th^ edition as its start point and this was applied through to its completion. Obviously this eight-year difference is important, works such as those of Brisson (1760) and Pallas (1764) were excluded. A second problem with this resource is the spelling of scientific names. Sharpe and some or all of his co-workers believed names should comply with their understanding of the rules of Latin and Greek grammar and they made many corrections. In the majority of cases original spellings are faithfully reproduced in the citations in the helpful synonymies for each taxon discussed, which draw on most of the relevant books, but there are certainly some cases where the spellings here were also emended. These emendations seem to be the single major reason why ornithology has suffered from competing spellings. In spite of these two problems, the *Catalogue* contains a huge bedrock of knowledge. However, almost all the volumes appeared before Sherborn (1902). Thus, for example, the name *Strix barbata* Latham, 1790 (see Sherborn 1902: 108) was missed by Sharpe (1875) which has led to the name being almost invariably attributed to Pallas (1811). Sharpe’s five volume *Handlist of the Genera and Species of Birds* (1899–1909) is a useful summary of the *Catalogue*.

In the early 20^th^ century there was a general understanding of the value of such lists and of the need to keep them up to date. By the 1920s it was apparent on both sides of the Atlantic that much new information had been accumulated and Sclater (1924: [iii]) wrote:

“The scheme for the publication of a systematic list of the Birds of the World, according to Zoogeographical Regions, had its origins in a proposal laid before a Committee of the British Ornithologists’ Union in 1919, when a special committee was appointed to take the matter into consideration.” “The Committee have [sic] held many meetings, and have been in communication with the Secretary of the American Ornithologists’ Union, and an agreement has been reached in conjunction with that Union in the preparation of the Lists of the Birds of each Zoogeographical Region, the B.O.U. being responsible for those dealing with the Old World.” This led to coverage of the Ethiopian Region (Sclater, 1924, 1930) and the Australasian region (Mathews, 1927, 1930 and supplements), but not to global lists for the bulk of Asia, nor to a Palaearctic list. In the case of the latter, Ernst Hartert’s *Die Vögel der paläarktischen Fauna* (1909–1934) was filling the gap and no work in English was started.

In America work had already begun on the *Catalogue of birds of the Americas and the adjacent Islands in the Field Museum of Natural History including all species and subspecies known to occur in North America, Mexico, Central America, South America, the West Indies, and islands of the Caribbean Sea, the Galapagos Archipelago, and other islands which may be included on account of their faunal affinities*. Of this 15 volumes appeared between 1918 and 1950, the authors being Charles Barney Cory (1857–1921), Carl (Charles) Eduard Hellmayr (1878-1944) and (Henry) Boardman Conover (1892-1950).

Although the above catalogue was still unfinished it gradually became evident that the coverage of Asia and the Palaearctic was either non-existent or dated. Perhaps it was the need to deal with these gaps, or just the obvious value of having all the birds of the world in one reference work in the English language, that led to the initiative to do just that. The new conception, and the second key source for material for avian synonymies, was the *Check-list of Birds of the World*, begun by James Lee Peters (1889-1952) in 1931. This 15-volume work emanating from Harvard University was completed (except for the sixteenth volume holding the General Index) by the publication of volume 11, in 1986, 34 years after the death of Peters. It soon became *the* standard work, but only volume one was ever updated. The completion of this after the death of Peters was due to the work of a variety of internationally-known ornithologists led and encouraged by Ernst Walter Mayr (1904–2005). This was a period when the perception was that many species were mere local variants and the merging of species, with little or no pre-publication of reasons, was common. A similar compression of genus-group names occurred; many became synonyms or hidden subgenera.

From the beginning it was Peters' intention that names listed in synonymy in the above mentioned *Handlist* and still considered synonyms would not be re-listed – in spite of the well-established use, by now, of subspecies. Over the years that intention was held to, but deliberate omissions of synonyms began due to other major works, but regional, not global ones.

In the Introduction to volume I of his *Check-list* Peters (1931: [v]) wrote “It is now nearly thirty-two years since the first volume of Sharpe’s *Handlist* .... made its appearance. The five volumes comprising that work have long been the one and only standard catalogue available to ornithologists, and it is a pity that Sharpe’s work could not have remained so, but the rapidity in the increase of ornithological knowledge has made it clear for a number of years that a new work along the same, or perhaps slightly more elaborate, lines was needed”. One of the strengths of this new compendium was the listing of the type locality for each listed name. A few key points from that Introduction (p. vi) need reporting. “... it does *not* include a complete principal synonymy. Synonyms, both generic, specific and subspecific, are given only for genera, species and subspecies described since the publication of the first volume of Sharpe’s *Handlist*”. However, Peters (op. cit.) also wrote “Synonyms not to be found in any of the volumes of the *Catalogue of Birds of the British Museum* are cited in full”, which was slightly at odds with his earlier sentence and implied that names prior to 1766 had all been accounted for and were given, which is not strictly correct. With the appearance in 1960 of volume IX of Peters’ *Check-list of Birds of the World* Ernst Mayr and James Cowan Greenway Jr. (1903-1989) were appointed editors; and in fact it was Mayr in particular who guided the project to its completion. Volume IX was the eighth to appear because volume VIII had been delegated to John Todd Zimmer (1889-1957) and he had died five years after Peters leaving that volume unfinished. In the Introduction to volume IX Mayr and Greenway (1960: vii) revised the limitations to the included synonymy, writing “The synonymy of Old World taxa includes all names proposed since Sharpe’s *Handlist*, or, where appropriate, Hartert’s *Vög. pal. Fauna*, while synonyms of New World taxa correctly cited in Hellmayr’s *Catalogue* are not repeated.” Users of the second edition of Peters *Check-list* volume I, which dates from 1979, should note from its Introduction (p. vi) that Mayr and Cottrell (1979) stated “Synonyms correctly listed in the first edition have been omitted ...”. Because of these and other limitations it is clear that to derive full synonymies other works, and not just those mentioned above, need to be consulted.

As work on Peters’ *Check-list* seemed to head towards completion, the Introduction to volume X (Mayr and Paynter 1964: v) claimed leadership throughout the zoological world saying of the *Check-list* “There is nothing like it in the world literature for any other kind of organism.” However, the last volume to appear (Vol. XI, 1986), apart from the index (vol. XV, 1987), came out 22 years later. Whether or not this work led the world it remains of very great use to ornithologists for its content, especially its inclusion of type localities.

The Richmond card index may have been reasonably complete in 1930, but after that adding new cards depended on his successors. While some of these are known to have shared his enthusiasm for this resource there will certainly have been periods when a lower priority was assigned to this task. The *Zoological Record* should help with completion, but its journal coverage has never been as complete as its compilers would have wished. Finally, a few private individuals have been assiduous in collecting new names and their information will need to be obtained. However, it seems probable that since the 1990s no ornithologist has been paid to maintain a list, despite the fact that new species and subspecies of birds continue to be found and named every year and new genus-group names proposed. Amateurs, however, have done their best to fill the gap and the ‘*Howard and Moore complete checklist of birds of the world*’ edited by Dickinson (2003) made full use of their gatherings even if sometimes only in footnotes.

In the period from 1851 onwards, i.e., after the *Index Animalium*, scientific zoological journals begin to multiply and then specialise and the earliest ornithological journals had their beginnings about that date (*Naumannia* in 1849, the *Journal für Ornithologie* in 1853 and *The Ibis* in 1859). The cataloguing of zoological journals arrived earlier.

The *Royal Society Catalogue of Scientific Papers* covered the years 1800 to 1863 (and in a second series covered up to 1900); but the output in zoology was so large that in 1864 the Zoological Society of London launched *The Zoological Record*, now commercially published by Thomson Reuters. This provides separate listings for the literature of each class of zoology. Almost certainly everything reported in these lists will have been indexed by Richmond and his successors. Some tens of journals from smaller countries, with limited facilities for the study of zoology, have at times neglected to provide the indexers with their works and the extent to which new names have been missed is not certainly known, but the number we lack is probably quite small, say less than 2 or 3%.

An effort similar to that of the Royal Society was made in Germany where the *Archiv für Naturgeschichte* provided quality information from 1839 onwards on the main zoological subjects covering both books and periodicals. The sections on birds were called *Bericht über die Leistung in der Naturgeschichte der Vögel während des Jahres ....* and the successive compilers were Andreas Wagner, Gustav Hartlaub, August von Pelzeln and Anton Reichenow. When these ceased towards the end of the 19^th^ century Reichenow ensured that similar material appeared in the *Ornithologischen Monatsbericte* and indeed the primary journals (such as those mentioned above) that had arisen to serve ornithology all provided such information as they were able to collect and consider.

Peters (1934) was fulsome in his praise of the utility of the works of Sherborn and Richmond and also John Todd Zimmer (1889–1957) whose *Catalogue of the Edward E. Ayer Ornithological Library* appeared in 1926. Zimmer’s bibliographic work was paralleled by research by Gregory Macalister Mathews (1876–1949) published in 1925 as two supplements to his *Birds of Australia*, and followed later by an important and more detailed but unfinished work by Robert Morrow Mengel (1921–1990) of which two parts were published (A–B in 1972 and C–D in 1983). The complex part works of John Gould (1804-1881) were very carefully documented by Gordon Sauer (1982), who, like Mengel, used the important Ralph Ellis Library of Ornithology at the University of Kansas. Studies such as these were encouraged by Sherborn as a founder member of the Society for the Bibliography of Natural History. This has now broadened its interests and been renamed the Society for the History of Natural History.

A recent account of the various sources likely to be needed by bibliographers working in ornithology, but also important to those working in other zoological disciplines, is to be found in Dickinson et al. (2011). The foundations for ornithological nomenclature ultimately comprise the many books and periodicals published that made possible these and later compilations.

## What remains to be done?

In the 20^th^ Century it began to be said that we knew all the birds, as the rate of discovery of new species suggested to the public that accumulation of ornithological knowledge overall had plateaued. Peters (1931: v) had referred to the “rapidity of the increase of ornithological knowledge” since Sharpe’s *Catalogue*. But Zimmer and Mayr (1943) wrote “practically all the widespread species of the birds of the world have been discovered, whether they be rare or common. There still remain a number of tropical islands, mountain ranges, or isolated peaks on which additional new species will be discovered”. Mayr (1957) wrote “... I doubt that more than 20 new species will be discovered in the next 10 years”. However, with at least 35 new species discovered in the decade following that comment, Mayr later (1971) suggested that this rate of discovery might continue and indeed it has.

Ornithologists found that research into behaviour and ecology opened new frontiers, revealing new taxa, filling needs for conservation biology and supporting the interests of a growing community of bird-watchers who became more serious with increased leisure time and cheaper travel. This was also supported by the introduction of field guides with good colour plates and an increasing willingness by publishers to depict every species in colour (Moss 2009). Television further popularised birds and they are clearly among the star turns that support interest in, and fund-raising for, conservation.

More recently phylogenetic studies of birds have formed the vanguard of evolutionary biology. Stresemann (1959) lamented that 200 years since Linnaeus we still lacked a reliable phylogenetic framework for the relationships of birds. However, the discovery of the structure of DNA in 1953 created new opportunities for molecular approaches to phylogenetics, seized upon by ornithologists. Charles Sibley’s comparative data on egg white proteins are recognised as game-changing for building molecular phylogenies. Sibley (1965) wrote “we have studied ... all 27 orders and ... 146 of the 170 families of living birds” and followed this with DNA-DNA hybridization work leading to the first avian checklist built on inferred phylogenetic relationships (Sibley and Monroe 1990). Molecular biology of birds remains at the forefront of both academic and applied research, with the extraction of DNA from blood, feathers, bone or tissue of live and dead birds now commonplace. We have been finding, first, that many perceived relationships inferred from anatomy and morphology have been over-simplified and, second, that we have misperceived the diversity and need more genera and families to sensibly structure avian diversity. Names help structure knowledge. The 170 families mentioned by Sibley contrasts with today’s checklists of 100 non-passerine families plus 136 passerine families (Dickinson and Remsen 2013; Dickinson and Christidis 2014): a 39% increase in just under 50 years.

Currently, tools for disambiguation of bird taxonomy are developing that also include nomenclatural links. There is a close working relationship between the authors of the 2013-14 checklists (Dickinson and Remsen 2013; Dickinson and Christidis 2014) and Avibase (Lepage et al. 2014 and http://avibase.bsc-eoc.org/), a large taxonomic reconciliation, distribution and current names resource. The Avibase compiler is the database manager for the checklist work and has approval to insert checklist content into Avibase as soon as the checklist is actually published.

This is where the access to nomenclatural information requires an upgrade. Many of the genus-group names needed exist in synonymy but this is not immediately obvious due to the lack of detailed, organised synonymies – synonymies (which, so that date precedence can play its role, must give the authors and dates for the genus-group names mentioned) should clarify the type species and make clear which synonyms are objective, because they are based on the names of species already used for an earlier genus-group name, and which are subjective, and are listed where they are only because of a taxonomic judgment that the type-species concerned is satisfactorily placed within a broader genus. In fact, such synonymies would greatly improve the selection of species for taxon sampling because screening the type species of genus-names in synonymy allows more explicit results to be postulated.

Wilson (2005) set out a vision of rapid progress by the biological sciences with considerable expectations from molecular and cellular biology. In ornithology the number of publications based on molecular studies year is clearly growing. But, molecular studies are being increasingly complemented by evidence of behavioural traits, including song, that can be strongly suggestive of species limits, and indeed much recent change reflects anatomical and morphological evidence that had led to the recognition of extra genera that were swept into synonymy in the mid 20^th^ century.

**Table 1.** Recent published molecular studies in ornithology. Separate columns list papers of global relevance; those related to the “Old World” (including Australasia), and those related to the Americas based on the coverage of each study (source: Dickinson and Remsen 2013).

Year	Global	Old World	New World	Total
2001	23	18	37	78
2002	22	27	45	94
2003	29	27	49	105
2004	42	43	45	130
2005	28	44	56	128
2006	33	31	67	131
2007	32	62	69	163
2008	40	59	86	185
2009	44	48	68	160
2010	39	62	98	199
2011	50	61	100	211
2012	36	71	92	199

There is no detailed published synonymy of avian genus-group names of the kind explained above. We have some 10,000 species of birds and over the years since 1758 perhaps double that number of genus-group avian names has been proposed. As only about 2500 of these names are in current use, it seems that each genus name we employ must have about seven synonyms! Molecular biologists researching birds need a comprehensive synonymy of avian genus-group names; one where they can determine what names, within a broad genus, are found in its synonymy and how some of these names will dictate any subdivision of that genus. Of course it will sometimes be the case that a genus including two or more sections, which appear in the phylogenetic tree as clades, lacks a name in synonymy that is representative of and applicable to each clade. In such cases new generic names will be needed. However, if the genus or family under study is ‘mapped out’ in terms of its synonymy and the taxon sampling includes the type species of the genus-group names in synonymy, the taxonomic evaluation of the results will be making optimal use of nomenclatural structuring of knowledge. This will help reduce the need for corrections to changes proposed without full evaluation.

This serious lack of full synonymies is, I suggest, partly due to how few alpha-taxonomists are now paid to do such work, but there is also a generalised failure in ornithology to recognise this need and to collaborate internationally, and to use the revolutionary tools offered by digital information systems to create databases such as synonymies. The vision shown by Sherborn, Richmond, Peters, Mayr and others has not been sustained in a world where ornithology is organised without a sufficient consideration of prioritisation of resources and without central direction for international projects other than those in conservation. Ornithology is largely organised in national societies, with limited terms of elected office and those taking office at such levels are the willing and the available, and are rarely the long-term thinkers and the visionaries. Behaviourally such office-bearers are more like politicians: they have a shorter-term focus. An integrated approach to taxonomy such as that suggested by Padial et al. (2010) will simply not materialise without the creation of expert groups and determined delegation to them by such elected officials. This already often occurs at the national level but it is not visibly working at the international level.

Other zoological disciplines have managed data collection and organisation much better. Ichthyology for example has “Fishbase” (http://fishbase.org). It is interesting and relevant that this was largely an unfunded labour of love, with a key founder, Bill Eschmeyer, but that there was also a huge cooperative research community. The value of “Fishbase” to all fish workers, in all aspects of research and applied work, is widely agreed (Pyle 2015). There are some ornithologists who also perform such labours of love; however all, or almost all, are amateurs working individually. Unfortunately the ornithological community has not pulled together in the same way as the fish folk! Although the sheer volume of the bird literature is a challenge, it is certainly a surmountable one with distributed, collaborative effort. The bird community should make it a priority to produce a similar resource over an appropriate time period.

There is now some potential for collaboration. What was the Standing Committee on Ornithological Nomenclature, established by the International Ornithological Congress has, in 2015, morphed into the Working Group on Avian Nomenclature of the International Ornithological Union. The change has led to an increased number of members and to recognition that it must involve itself in the challenges in producing Lists of Available Names. What is unclear is how much can be achieved in a timely manner without financial support.

If there is a problem with a lack of a source of synonyms, now apparent at the generic level but certainly suffered at the species and subspecies level, this also affects names at the family level. Bock (1994: 159) mentioned his difficulties in examining some of the very early works in which he sought for the reasons for changes in usage of generic names (in the cases where such names provide the stems for family group names). This is a complex problem; most of us will not have total recall and not know where to find crucial evidence, in addition actually locating some older works and gaining access has been a problem. Since Bock’s period of study much has changed and a growing body of such works is available through the Biodiversity Heritage Library (www.biodiversitylibrary.org). Our museum librarians, it seems, do have the vision needed and have identified ways to fund a tremendous resource – benefitting especially the smaller countries of the world with limited local library facilities and also the amateur working at home with limited resources.

The value of bringing together data that relates to particular taxonomic groups is increasingly recognised; scientific names and common names, in all languages, are being linked in “name-use catalogues” or indexes by organisations such as GBIF (the Global Biodiversity Information Facility – www.gbif.org). However, as identical names are used for taxonomic concepts that are not identical (e.g. for a broad species with perhaps 10 subspecies or for the nominate subset of that which follows a separation into two or more species) there is an increasing need for taxonomists to mediate the understanding of what such aggregations of data tell us. This is where the lead taken by Avibase is important because within Avibase each such concept is mapped so that the scope of each concept attached to one and the same name and can be seen clearly. Nine different taxonomic authorities can be compared within this resource, which contains 14 million records of about 10,000 species and 22,000 subspecies of birds.

## Filling the gap – the efforts made so far

The compilation of institutional indexes or databases of avian names, such as the card index developed by Richmond at the Smithsonian Institution or a similar one for recent avian names maintained by the Department of Ornithology of the American Museum of Natural History, New York (AMNH) until the late 1990s, have probably all been discontinued due to pressures on personnel and a focus for computerisation on collection holdings and thus data-capture focussed on specimen registers.

However, such card indexes have proved their value. Drawing on the AMNH card index, and with extra inputs from Norbert Bahr, he and I developed a list of new avian names since the volumes of Peters’s *Check-list*, which, by 2001, allowed the 2003 edition of the *Howard and Moore complete checklist of the birds of the world* (Dickinson 2003) to mention every name then known to have been proposed since Peters *Check-list*, to which it became a vital companion work. No other recent global ornithological checklist had previously set out to do this. All the subspecies names in that 2003 checklist were supplied to Alan Peterson, and he now has the best-organised database of ornithological names known to me (although it is incomplete). He makes this available on www.zoonomen.net.

Alongside his database, which is focussed on names in use, Peterson displays scans of all the cards of the principal card index developed by Richmond thus providing information on the both original citations of names in use and of synonyms that found their way into Richmond’s card index.

Because of both the relative completeness of Peterson’s data and the way he has it organised it, this data should be the basis for preliminary population of ZooBank with avian names. He already makes his content available to ITIS (the Integrated Taxonomic Information System – www.itis.gov) and the Encyclopedia of Life (www.eol.org). However, only in ZooBank is it seriously likely that eventually there will be carefully-structured validation of all these names; and also ZooBank is perhaps the most logical repository to promote to encourage ornithologists to strive to create for comprehensive coverage at an even higher level, for example by including family-group names. This is a collaborative task and it should be complementary and enabling for LANs (Lists of Available Names).

As in other zoological fields, there remain bibliographic problems that have not been resolved. Dickinson et al. (2011) listed and discussed 148 books containing new names in ornithology (and sometimes other fields) that have presented, and in some cases still present, problems of accurate dating. Although such cases were often explored by Sherborn, or other later authors, evidence unknown to them is still being found and changing our understanding of the correct dates to attribute to taxon names. Detective work in this field can be very rewarding. I explain briefly below four cases where recent studies have either caused dates to change (or to be retained – although known to be wrong).

The *Nouveau recueil de planches coloriées* of Conraad Temminck and Meiffren Laugier (1820-1839): Sherborn (1898) made us aware that of the 101 parts of this work the first twenty initially lacked texts and that in these cases because the plates gave only French vernacular names it was necessary to find the wrappers of each part to discover, and be sure of, the spelling of any new original name. Wrappers are the encasing parts produced separately but to be grouped for binding (sometimes obviously based on pagination, sometimes in a format instructed later). These often include specific publication information that is not included once the volumes are bound. Wrappers may include the only precise indications of publication dates and thus can be very useful in bibliographic detective work. Of these twenty wrappers he knew of just two. Over a century later Dickinson et al. (2011) knew of no more, but later the same year all the remaining wrappers began to surface and they were photographed and published (Lebossé and Bour 2011, Dickinson 2012). However, another problem plagued this work. Temminck had promised that each part would contain six plates but 600 plates, the full complement, required 101 parts to be published and which parts had fewer than six had not been determined so that numerous citations were potentially wrong. This riddle was resolved by Dickinson (2001), and proved by Temminck’s handwritten list in the archives of the museum in Leiden, meaning that each plate is now associated with just one part and thus date of publication.

The monograph on pigeons and doves with text by Temminck and plates by Pauline Knip (née de Courcelles) in 1808–1811: although it was generally known that the artist had in some way made herself appear to be the mainspring of this work, exactly what happened and how this might affect dates of publication or citations was unclear. Here, evidence was available but it had not been compared and understood. The full details of what turned out to be a fraud were published by Dickinson et al. (2010) and several dates of publication had to be changed. It was concluded that Temmick (1808–1810) was the sole author of the first 13 livraisons (one of the numbers of a book published in parts) under the title *Histoire naturelle générale des pigeons* and that the last two livraisons, under the title *Les Pigeons* must be credited to Knip and Temminck (1811) (the latter alone being responsible for any new names).

The *Catalogue of the birds of the Peninsular of India* by Thomas C. Jerdon: began as serialised parts in the *Madras Journal of Literature and Science* but the [first] Supplement, in part 30 of that journal, was long delayed and we found that Jerdon had had a 200 page catalogue privately printed in 1840 or 1841 which included the published parts and the delayed part, and that this was three or four years before its formal appearance in the journal in 1844. The dates of several new names from that supplement were thus advanced to 1841. See Dickinson et al. (2004).

The *Histoire naturelle des oiseaux de l’Amerique septentrionale* of Vieillot: was discussed by Richmond (1899) who mistakenly believed, and seems to have led Browning and Monroe (1991) to believe, that the first part was published on 1 December, 1807. In fact what Richmond saw was merely an announcement that appeared four weeks after that saying it “will be published” on that date. A later notice in July 1808 changes the story and said that it “will be published” on 1 September 1808. The date of each part that was suggested by Browning and Monroe was a merely a projection of a very rapid, and almost certainly over-optimistic, timetable based on a start date that was at least nine months out. Indeed, based on the delay of the first part, it is extremely unlikely that subsequent issues followed on schedule every month. But here we have no correct dates to use and for the moment the incorrect dates are retained to avoid premature changes to nomenclatural stability (Dickinson 2011).

Of the many periodicals also discussed by Dickinson et al. (2011) the two most complex problem cases appear to be the *Journal für Ornithologie* and the *Proceedings of the Academy of Natural Sciences, Philadelphia*. The dates of many of the early issues of both are doubtful, because some issues are known to have been delayed. A complete picture of which issues were delayed and which were published on time requires detailed painstaking work and may well still be far from resolution. Numerous other periodicals, with fewer new avian names in them, also await serious review.

**Figure 2.**
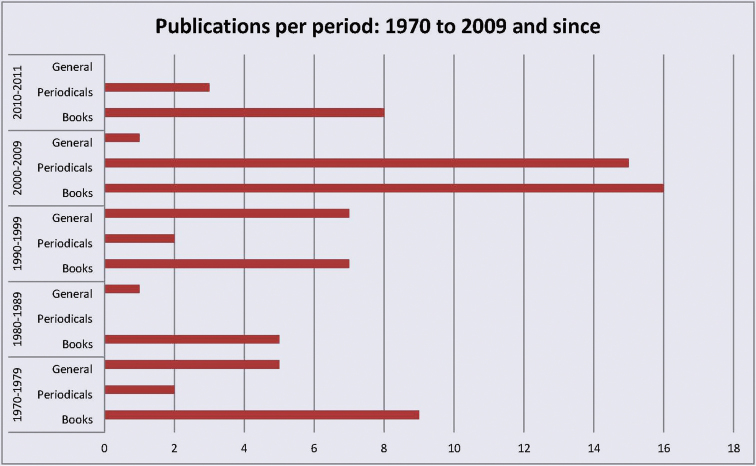
Bibliographic publications per decade, 1970 to late 2011, by subject. Treating books, periodicals and general works such as catalogues (source: references cited by Dickinson et al. 2011).

Articles on subjects like these were quite plentiful in the early years of the *Journal of the Society for the Bibliography of Natural History* (now the *Archives of Natural History*), up to approximately 1980, by authors such as C.E. Cowan, F.J. Griffin, M. Guédès, F. Hemming, L.G. Higgins, M.E. Jahn, R.I. Johnson, W.L. McAtee, N.F. McMillan, H.S. Marshall, E.C. Nelson, J.H. Price, F.C. Sawyer, C.D. Sherborn, W.T. Stearn, J.C. Thackray, A.C. Townsend, A. Wheeler and P.J.P. Whitehead (names sourced from the list of authors in Nelson 2001), but unfortunately such detective work, ideal for retired zoologists with good bibliographic skills and an appetite for rigour, attracts few recruits these days. The decline in such publications between 1980 and 1989 is shown in Fig. 2.

However, there has been a resurgence of interest in the subject at the end of the millennium as zoologists realise that digital tools can make this kind of bibliographic foundation easier to build and more important than ever for the age of biodiversity bioinformatics. In this period undoubtedly the most influential author has been Neal Evenhuis (e.g., Evenhuis 2003a, 2003b, 2008), but others such as Kraig Adler, Kees Rookmaaker and Florence Pieters have contributed, as has the present author. Lyal (2016) outlines how legacy zoological literature should be most effectively digitised and utilised.

## Filling the gap – what remains to be done

Looking to the future and assuming that ZooBank becomes the long-term solution to finding all avian names, this will require its population, verification of such retrospective imported records and a programme to complete the entries of old names. This programme might well be started as part of the development of Lists of Available Names (LANs). I suggest first a LAN for avian family names, then for avian genus-group names and eventually for species-group names. This is being facilitated by the dissemination of full guidelines for the submission to the I.C.Z.N. of such lists, and for their consideration and potential adoption (Alonso-Zarazaga et al. 2016).

Bock’s (1994: 121–123) reservations relating to the previous Code are still of relevance to these guidelines. In a nutshell, Bock argued that meeting the original expectations of the Commission required a wholly disproportionate time commitment. His six or more years of research led him to list 1400 names (although perhaps 15% of these need not be counted again as they reflect the operation of the Principle of Coordination, explained in Art. 36 of the Code). Much time was indeed committed, but it is not clear that this was disproportionate to what might have been expected or to the value of what he produced. Behind Bock’s observation was an acute awareness of the size of the corpus of serious ornithological literature, and it is fairly certain that that body has grown faster in the last 20 years. While much of the older, rarer, content may now be easier to access, thanks to BHL, some later works will be harder to access due to copyright restrictions.

In parallel, the ornithological community needs to set up and sustain a collaborative process to see that all new names do get added to ZooBank. However much encouraged, publishers and authors will not all register what they should unless registration becomes mandatory for a name to be validly published.

Discussions aimed at stimulating collaborative work on avian generic synonymies began over five years ago and numerous promises of help on specific families have been received and will be taken up! This suggests that this need is well understood and becoming more so due to the increasing quality of molecular studies and of the interpretations of their results. In addition, continued bibliographic research should be encouraged, and this is no doubt true for other fields of zoology so that interdisciplinary collaboration and data-sharing will be highly desirable.

**Figure 3.**
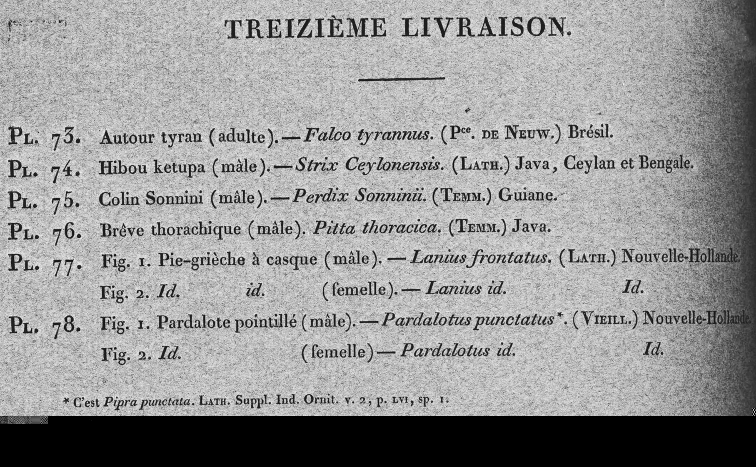
The wrapper for the 13^th^ part of the *Planches Coloriées* showing vernacular and scientific names. All the wrappers for the first twenty parts, all that were published without supporting texts, have been depicted in *Zoological Bibliography*: 10 in vol. 1, No. 4, pp. 144–148 and 10 in Vol. 2, No. 1, 37–41 pp. These pages were published under the terms of the Creative Commons Attribution Licence and may be reproduced with proper attribution.

The wrappers of books published as part-works, so often discarded when the work was complete and sent to the binder, are now often either completely missing or unrecorded. There is an enormous need to pool information on extant wrappers, at least for those critical to the dating of new taxa or to the spelling of their original names. Ideally we need illustrations of all such wrappers to be scanned for the Biodiversity Heritage Library (BHL), especially when the displayed content on the BHL website will be misinterpreted without such illustration. My favourite example is, of course, the “*Planches Coloriées*” of Temminck and Laugier, mentioned earlier. This is a special case because the first 20 parts (120 plates) appeared before the texts issued for them; no scientific names were on the plates, they appeared only on the wrappers accompanying each set of six plates (see Fig. 3). Happily all twenty wrappers have now been located, with the images of their lists of contents published (Lebossé and Bour 2011, Dickinson 2012) and are available for use by BHL.

The wording of Art. 12.2.7 of the Code implies that a combination of text on a wrapper and a plate linked to a vernacular name on both meets the requirements for valid introduction of a name before 1931, so that the new names in these wrappers date from the issue of the plates. In *Index Animalium* Sherborn was not consistent in dating names from the *Planches Coloriées*. Years earlier Sherborn (1898) provided dates for each part, building on what was clear from the weekly issues of the *Bibliographie de la France*, but in *Index Animalium* he did not consistently apply those dates to the names in the first 20 parts; quite often he used dates that appear to come from published findings relating to when the subsequent texts for when those parts appeared. The discovery of the full set of wrappers permits consistent recognition of the original spellings from the wrappers and encourages dating from their appearance and not from the date of issue of the later text. However, what Sherborn did here may suggest that there are problems with the dating, in *Index Animalium*, of other older books that appeared in parts (Welter-Schultes et al. 2015; Dickinson et al. in press). Again it becomes clear that all existing datasets have some problems and that verification of name-records in ZooBank will require triage, with complex works referred to expert bibliographers.

It is also desirable that explanations of the findings drawn from all sets of wrappers be published as recently done by Lyal (2011) in the case of the *Biologia Centrali-Americana*. The same is true for research into dates for runs of journals; see, for example, Poggi (2010).

As a community, we must also encourage the managers of the Biodiversity Heritage Library (BHL) to help. For example, when journals – or books which were part-works – are scanned, every effort should be made to locate wrappers and to scan and display these alongside the content. The wrappers found by Lyal (2011) are available at the Natural History Museum and could be scanned. The photographs of the Temminck and Laugier wrappers can be made available to the Biodiversity Heritage Library (BHL, http://www.biodiversitylibrary.org/) without charge. Others may need to be specially photographed. Ultimately, notices of what is lacking in this regard should be circulated widely to prompt institutional libraries to locate and offer rare material of this kind from their holdings for scanning.

**Figure 4.**
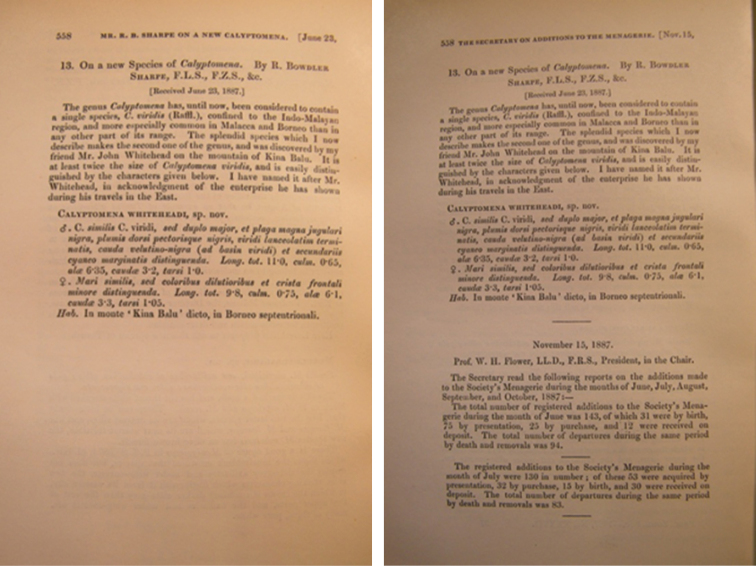
An example of two-state publication. Here the *Proceedings of the Zoological Society of London*, 1887, demonstrates a two-state situation. Left image: “first state”. Right image: “second state”. Images from Dickinson et al. (2011: 36) reproduced with permission from The Natural History Museum, London.

As regards journals, date research is easiest with sets in which the issue wrappers have been bound in (preferably in place rather than at the end of the volume). While the practice of binding these at the end is usually sufficient, it is unsafe to assume this is definitive. For many older journals issues did not actually end where the bound volume may suggest! Many journals that had a page or two of the final signature, of say eight pages, blank, later began the signature again at the start of the next issue so that pages would run on smoothly. In other words, some pages appeared in two “states”, one with empty space on the page and one with that space filled. Examining such pages in their second state can falsely convince the reader that part of an article appeared in the previous issue, see, for example, Dickinson (2004) as illustrated above (Fig. 4). But even this can be deceptive because sometimes some text not accommodated in the final signature first appeared on the back wrapper before re-appearing on the first page or so of the first signature of the next issue!

To the extent that the Code (ICZN 1999) provides rules governing dates of publications, it takes priority seriously in recognition of its importance both for synonymies and for the validity of a name when precedence is shown to be an issue potentially requiring change. However, these rules should be rather more detailed and in particular should be less ambiguous. Suggestions for consideration when the Code is revised will be found in Dickinson et al. (2011: 15–23).

Editorial concern for the provision by journals of accurate dates of publication has declined although it is not clear that there was good reason for change. In 1990 the *Ibis*, the United Kingdom’s senior periodical in ornithology, ceased the provision (annually in arrears) of day-dates of publication of the four issues per year. Since then, and prior to 2012 and the changed relevance of electronic publication, on more than one occasion release of a January issue, which included the introduction of the name of a new taxon, actually occurred in the previous December. One such case has been acknowledged editorially the other has not. Thus one can be backdated, but to backdate the other requires retained proof of a date of receipt. Other journals in ornithology have had similar lapses – perhaps when commercial publishers unfamiliar with the Code take over the production and publication. However, it is not apparent that commercial publishers are worse than associations or even institutions such as museums. What has changed is that most student zoologists get no teaching time on the subject of nomenclature and the Code, and nomenclature is seen as a tiresome inconvenience rather than a tool designed to promote international dialogue through provision of as much stability in nomenclature as taxonomic change will allow.

The opportunity to register in ZooBank would seem to provide for the accurate future determination of dates of publication of works from 1 January 2012 onwards. However, this will only be true if the precise rules published in 2011 (ICZN 2011) are fully respected; in addition it will depend on an involvement by the publisher. In the case of retrospective registration it will be essential that there is provision for a date to be corrected and this may not happen during basic validation so allowance must be made for research results to be considered later and, when convincing, for them to be taken into use in ZooBank.

## Summary and conclusion

The tools that will facilitate needed future work are essentially available as a normal part of the array of digital programmes (e.g. web tools, spreadsheets and databases). However, they will certainly need some elaboration. Ornithology as a whole must take a look at itself and determine how collaboration can be mobilised and put behind such work. If this remains the task of a few individual enthusiasts then the challenges described here will not be met any time soon. Because nomenclature is the unsung foundation for taxonomy, it is taxonomic work, and thus accurate description of the living world, that will then ultimately suffer.
